# Paleopteran molecular clock: Time drift and recent acceleration

**DOI:** 10.1002/ece3.70297

**Published:** 2024-09-18

**Authors:** Soichi Osozawa, André Nel

**Affiliations:** ^1^ Institute of Geology and Paleontology, Faculty of Science Tohoku University Sendai Japan; ^2^ Institut de Systématique, Évolution, Biodiversité (ISYEB) Muséum National d'Histoire Naturelle, CNRS, Sorbonne Université, EPHE, Université Des Antilles Paris France; ^3^ Present address: KawaOso Molecular Bio‐Geology Institute Sendai Japan

**Keywords:** atmospheric CO_2_, BEAST v1.10.4, carboniferous, exponentially increased base substitution rate, fossil and geological event calibration, glacier period, Odonata, quaternary, tMRCA; quaternary and pre quaternary calibration

## Abstract

Applying BEAST v1.10.4, we constructed a Bayesian Inference tree comprising 322 taxa, primarily representing Paleoptera (Odonata and Ephemeroptera; Pterygota), Zygentoma and Archaeognatha (Apterygota; paraphyly), and Neoptera (Plecoptera; Pterygota), based on a 2685 bp sequence dataset. Our analyses revealed that robust dating required the incorporation of both Quaternary and pre‐Quaternary dates. To achieve this, our dating incorporated a 1.55 Ma (Quaternary) geological event (the formation of the Ryukyu Islands) and a set of chronologically well‐founded fossil dates, spanning from up to 400 Ma (Devonian) for the stem Archaeognatha, 320 Ma (Carboniferous) for the crown of Paleoptera, 300 Ma (Carboniferous) for the crown Ephemeroptera, and 280 Ma (Permian) for the crown Odonata, down to 1.76 Ma (Quaternary) for *Calopteryx japonica*, encompassing a total of 22 calibration points (events: 6, fossils: 16; Quaternary: 7, pre‐Quaternary: 15). The resulting dated tree aligns with previous research, albeit with some dates being overestimated. This overestimation was mainly due to the lack of Quaternary calibration and the exclusive dependence on pre‐Quaternary calibration, though the application of maximum age constraints also played a role. Our minimum age dating demonstrates that the molecular clock did not uniformly progress, rendering rate dating an inapplicable approach. We observed that the base substitution rate is time‐dependent, with an exponential increase evident from around 20 Ma (Miocene) to the present time, exceeding an order of magnitude. The extensive radiation and speciation of Insecta and Paleoptera, potentially resulting from the severe climatic changes associated with the Quaternary, including the commencement of glacial and interglacial cycles, may have significantly contributed to this increase in base substitution rates. Additionally, we identified a potential peak in base substitution rates during the Carboniferous period, around 320 million years ago, possibly corresponding to the Late Paleozoic Ice Age.

## Graphical abstract

Constant evolution rates, which form the foundation of the strict molecular clock model, do not apply in the case of Paleoptera, highlighting a notable bias in the molecular clock hypothesis. The current biodiversity may be the outcome of a relatively recent, exponential increase in base substitution rates. This could be a reflection of the initiation of Quaternary glacial and interglacial cycles linked to substantial climatic changes. Additionally, another peak was identified in the Carboniferous to Permian period, which is similarly associated with the late Paleozoic icehouse.

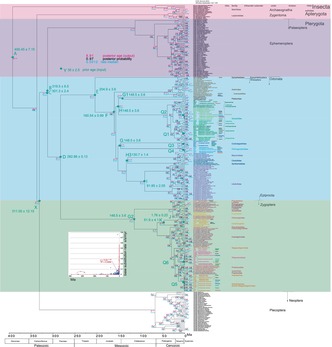

## 1 INTRODUCTION

**FIGURE 1:**
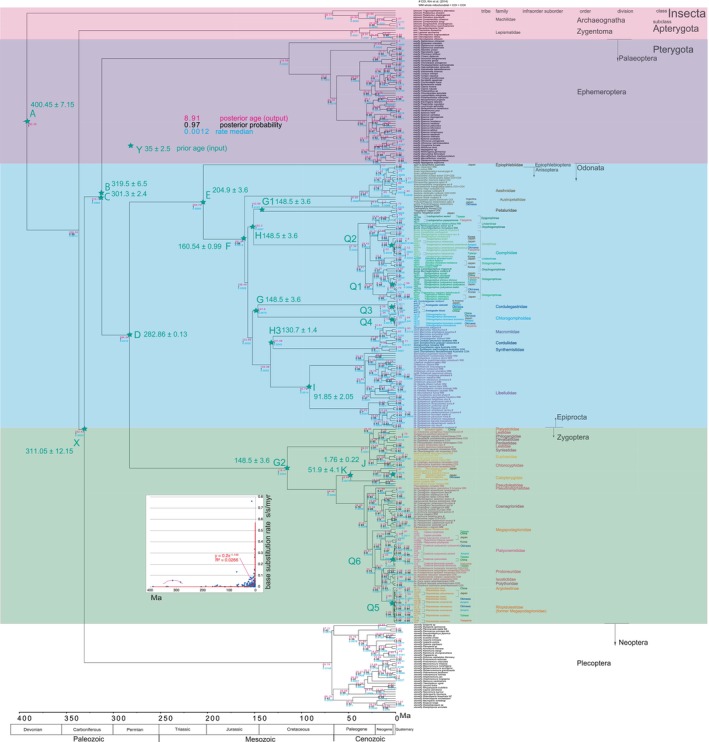
Bayesian Inference tree calibrated using both older fossil dates and the Quaternary geological event date with the aid of BEAST v1.10.4. Calibration points are represented by star marks and are detailed in Table 3. The calibration points labeled from A to K, and J (Quaternary) and X are based on fossil calibrations, while those marked Q1 to Q6 correspond to Quaternary geological event calibrations, specifically at 1.55 ± 0.15 Ma. For instance, Calibration Point I denotes the crown node of a common ancestor of the ingroup species of Libellulidae, but the stem node can be specified if necessary. In BEAUti, it is possible to specify a monophylum (clade) for Libellulidae ingroup species. It is important to note that the ingroup of geological calibration point Q6, for example, includes both Chinese Platycnemididae species and Ryukyu‐Taiwan endemic species, as shown in Figure 3. Notably, some sequence data were incomplete or not whole mitochondrial. Marked as WM are sequences with complete whole mitochondrial data, including both COI‐3P and COII‐5P, as exemplified by data from Wang et al. (2021). In cases where the data are not entirely whole mitochondrial, available COI‐3P, COII‐5P, or both are indicated (marked in). For instance, Kim et al. (2014) included 43 taxa from Korea, and while COI‐3P data are available, COII‐5P data were not presented (marked #). In addition, mitochondrial 16Sr RNA data for Japanese taxa were complete, as submitted by Ozono et al. (2012). Sequence data retrieved from Carle et al. (2015) and Ware et al. (2014, 2017, concatenated gene) were incomplete and largely unavailable for the present BEAST v1.10.4 analyses (combined gene). Specifically, COI‐3P and/or COII‐5P data were lacking for species including *Neopetalia punctata* (Neopetaliidae, South America), *Hemiphlebia mirabilis* (Hemiphlebiidae; Australia), *Heliocharis amazona* (Dicteriadidae, South America), and *Oligoaeschna* (*Sarasaeschna*) *pryeri* (Japan). The Plecoptera (stonefly) data were sourced from Zhao et al. (2020). Inserted figure: Base substitution rate versus age diagram. This figure illustrates the base substitution rate over time. The thick red curve represents a power trendline, accompanied by its equation and an *R* ^2^ value of 0.0266. The red thin curve depicts the expected approximate curve around an estimated age of 320 Ma (Carboniferous).

The recent trend in phylogenetic studies may be leading to larger tree and genome sizes, potentially resulting in more precise and detailed phylogenetic trees (Table 1). However, while increased sizes can enhance the robustness of topology, they do not guarantee strict dating accuracy. In other words, tree and genome sizes alone cannot calibrate the phylogenetic tree and are therefore inadequate for dating purposes (cf., Cicconardi et al., 2023). We perform node dating robustly and present a well‐established Bayesian Inference (BI) dated tree of Paleoptera (Figure 1), examining its implications, with a specific focus on the methodological approach of correlating base substitution rates with time, similar to Osozawa (2023). BEAST v1.10.4 includes a feature that allows for the visualization of base substitution rates and ages on each node (c.f., Cicconardi et al., 2023), facilitating the exploration of time dependencies (Figure 1 inset).

**TABLE 1:** Dated tree compilation.

References	Target	Tree size	Genome size	Application	Reference	Calibration point
Misof et al. (2014)	Incecta (mostly)	144	413,459 amino acid sites	BEAST v1.8	Drummond et al. (2012)	37
Montagna et al. (2019)	Incecta (mostly)	141	220,615 aligned amino acids	MCMCTree in PAML (4.4e)	Yang (2007)	43
Kohli et al. (2021)	Odonata	116	824,783 aligned amino acid sequences	MCMCTree in PAML (4.9 g)	Yang (2007)	17
Suvorov et al. (2022)	Odonata	85	2,167,861 aligned RNA sites	MCMCTree in PAML (4.9 h)	Yang (2007)	20
Legendre et al. (2016)	Dictyoptera	793	3674 bp	MrBayes 3.2.1 (r8s)	Ronquist et al. (2012)	17
Cicconardi et al. (2023)	Heliconiinae	63	4,011,390 bp	MCMCTree in PAML (4.8a)	Yang (2007)	4
present paper	Paleoptera (mostly)	327	2685 bp	BEAST v1.10.4	Suchard et al. (2018)	22

In the earlier phase, a molecular clock model could estimate the age of a tree by assuming a relatively constant base substitution rate. With this assumption, a dated phylogeny could be obtained using “the rate dating method” (MrBayes v3.2.7; Ronquist et al., 2019). For instance, a rate of 0.0115 substitutions per site per million years (s/s/my) was established for adaptive radiated *Heliconius* butterflies and is considered standard (Brower, 1994). Papadopoulou et al. (2010; MrBayes 3.1.2; Ronquist & Huelsenbeck, 2003; BEAST 1.4.8; Drummond & Rambaut, 2007) adjusted the COI rate to 0.0177 s/s/my and the nuclear 28S rRNA rate to 0.0006 s/s/my. These adjustments were made based on the preferred partitioning scheme and substitution model chosen using Bayes factors, and were calibrated applying the node age of the Miocene geological event for the Aegean islands (c.f., Ho et al., 2011), as applied to Tenebrionidae beetles.

Takenaka & Tojo, 2019; BEAST v.1.7.1 (Drummond et al., 2012) incorporated this rate into their BI tree analysis of Japanese mayflies, and Kanke et al. (2021; BEAST 2.5.0; Bouckaert et al., 2014) did the same for their BI tree analysis of Ryukyu endemic damselflies. Osozawa, Sato, et al. (2017; BEAST v1.8.2; Drummond et al., 2012) obtained a COI rate of 0.0607 and a nuclear 28S rRNA rate of 0.0205 for Ryukyu endemic damselflies, based on the Quaternary geological event calibration (1.55 ± 0.15 Ma; Osozawa et al., 2012).

**FIGURE 2:**
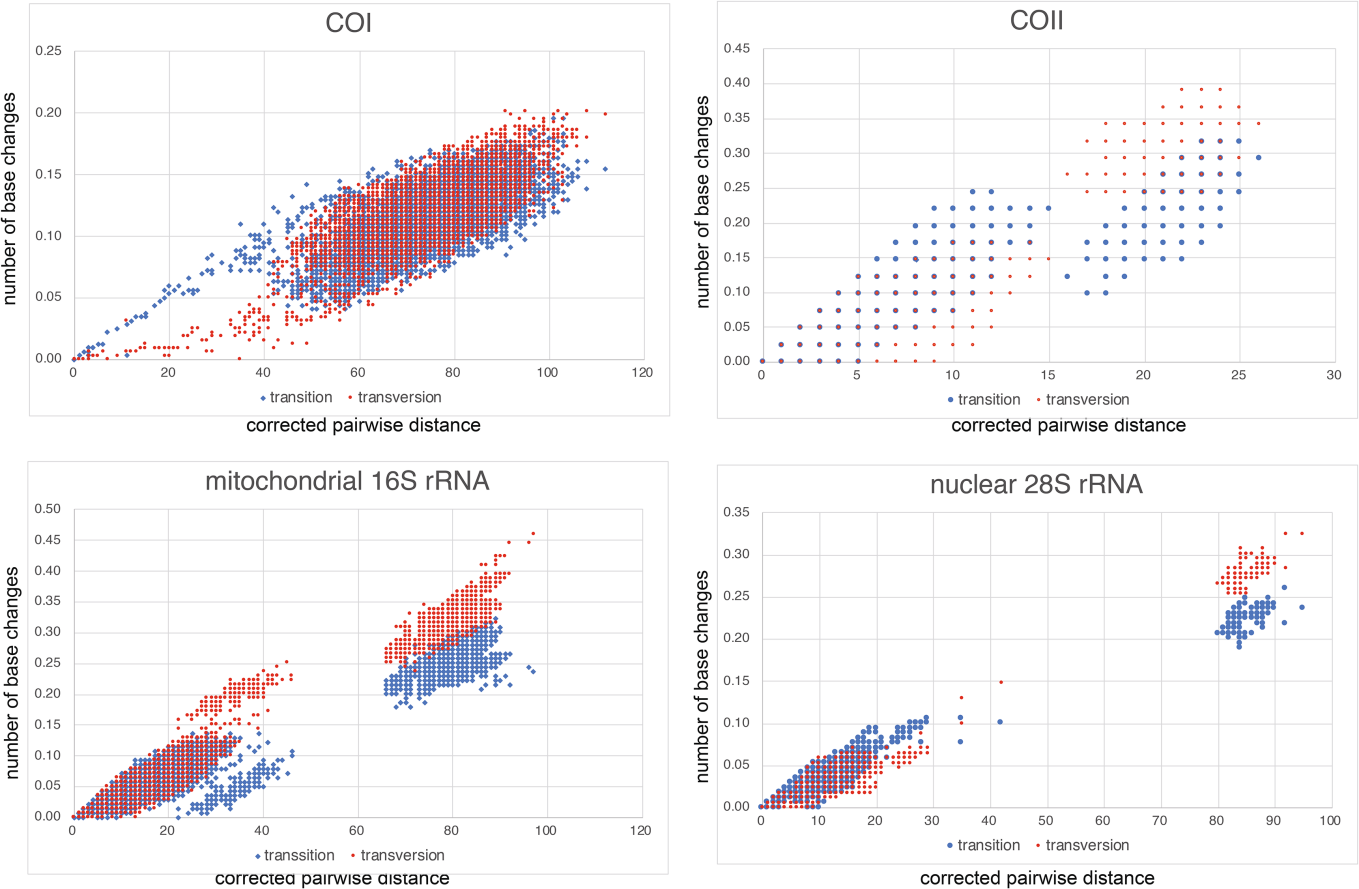
Number of base changes in transition and tansversion versus corrected pairwide distance diagram for mitochondrial COI, COII, and 16S rRNA, as well as nuclear 28S rRNA. In MEGA 11, we considered the first and second codon positions and excluded the third codon position following the methodology of Yuan et al. (2022; MrBayesv3.2.6; Ronquist et al., 2012) and referred to the approach outlined in Bi et al. (2023).

Especially concerning sister‐related species, it is crucial to note that in the maximum likelihood (ML) tree, each branch length, representing the number of substitutions per site, is distinct (e.g., see figure 2 in Osozawa, Sato, et al., 2017). This variability indicates that the base substitution rate progresses differently, rendering the application of the molecular clock hypothesis, or rate dating less stringent. One potential solution for molecular dating is to consider a relaxed clock model instead of a strict clock model (Zhang & Drummond, 2020), and dating applications offer the option to select the relaxed clock.

Molecular evolutionary rates, as seen in primate mitochondrial genes, have been observed to display both high short‐term (1–2 million years) mutation rates and low long‐term substitution rates, with the latter possibly attributed to purifying selection (Ho et al., 2005, 2011; BEAST v.1.3; Drummond & Rambaut, 2003). This time‐dependent nature of the molecular clock can influence a span of up to 1 million years (Papadopoulo et al., 2010). Although these studies have identified an increased intra‐specific genealogical rate during the Quaternary period, they received limited attention, with the exception of the works of Osozawa and Wakabayashi (2022; latest version 2023; BEAST v1.10.4; Suchard et al., 2018) and Osozawa (2023; BEAST v1.10.4).

In our pursuit of revisiting the time‐dependent phenomenon of increased base substitution rates during the Quaternary period (Ho et al., 2005; Papadopoulo et al., 2010), we have leveraged the Quaternary calibration date of 1.55 ± 0.15 Ma provided by *Coeliccia* damselflies endemic to the Ryukyu Islands (Osozawa et al., 2012; Osozawa, Sato, et al., 2017). We applied the same calibration method to endemic Platypleura in a global cicada BI tree (Osozawa, Shiyake, et al., 2017; Osozawa & Wakabayashi, 2022, updated in 2023). In this paper, we extend this approach by incorporating an additional five Ryukyu endemic Odonata species, all of which make use of the same calibration date of 1.55 ± 0.15 Ma (Figure 1; Q1–Q6).

Osozawa and Wakabayashi (2022) focused on Cicadoidea, including Tettigarctidae (Hemiptera; Neoptera). In this paper, our focus is on Paleoptera, specifically Ephemeroptera and the primary subject, Odonata. In the Insecta classification, we find the paraphyletic group referred to as “Apterygota” and the clade Pterygota, and as outgroups, we have incorporated the “apterygotan” Archaeognatha (jumping bristletail) and Zygentoma (silverfish).

Pterygota includes Palaeoptera, which lacks the ability to fold the wings back over the abdomen, in contrast to Neoptera (Ishiwata et al., 2011). Additionally, we have included Plecoptera (stonefly) as one of the primitive groups of Neoptera, based on research by Misof et al. (2014) and Montagna et al. (2019).

Our primary focus in this study is on modern Odonata, which can be divided into two suborders: Epiprocta (also known as Epiproctophora, encompassing damsel‐dragonflies and dragonflies) and Zygoptera (which includes damselflies). The extant Epiprocta suborder is further divided into infraorders: Anisoptera (comprising dragonflies) and Epiophlebioptera (formerly classified as the suborder “Anisozygoptera,” considered an intermediate group between dragonflies and damselflies; Bechly, 1996; Lohmann, 1996; Nel et al., 1993).

*Epiophlebia superstes*, a species belonging to Epiophlebioptera within the Japanese Islands, is a representative species. Historically, it has been considered a “relict” species (Asahina, 1954; Busse et al., 2012, 2015; Hasegawa & Kasuya, 2006; Ishida et al., 1988; Ozano et al., 2012; Suvorov et al., 2022; Wang et al., 2014).

## 2 METHODS

### 2.1 Materials

The total number of taxa in our analysis is 327, which is comparable to that of recent related studies listed in Table 1.

In our research, we included eight Archaeognatha, six Zygentoma, 57 Ephemeroptera, 217 Odonata, and 39 Plecoptera taxa. We ourselves specifically collected and analyzed 88 Odonata taxa (58 taxa in Table 2, with additional 30 taxa sourced from Osozawa & Wakabayashi, 2015, and Osozawa, Sato, et al., 2017). Of these 88 taxa, 53 taxa were endemic mostly in the Ryukyu Islands, and we applied a calibration date of 1.55 ± 0.15 Ma to these vicariantly speciated taxa (Osozawa et al., 2012). The remaining 129 sequence data (217–88) were obtained from GenBank/DDBJ. For additional taxonomic information, including accession numbers, refer to Table 2, Osozawa and Wakabayashi (2015), and Osozawa, Sato, et al. (2017).

**TABLE 2:** Odonata species and the corresponding accession numbers.

Isolate	Country	Species	Accession number mt COI	Accession number mt COII	Accession number mt 16S rRNA	Accession number nuc 28S rRNA	Collection date	Collected by
sg2	N Taiwan:Wulai	*Leptogomphus sauteri*	LC546150	LC546210	LC546270	LC546330	31‐Jul‐12	Soichi Osozawa
sg3b	Japan: Ryukyu, Ishigaki‐jima, Miyara‐gawa	*Leptogomphus yayeyamensis*	LC546151	LC546211	LC546271	LC546331	07‐Jul‐10	Soichi Osozawa
sg13	S Taiwan:Hualien	*Leptogomphus sauteri*	LC546152	LC546212	LC546272	LC546332	03‐Sep‐12	Soichi Osozawa
sg17	Japan: Ryukyu, Iriomote‐jima, Urauchi‐gawa	*Leptogomphus yayeyamensis*	LC546153	LC546213	LC546273	LC546333	23‐Jun‐10	Soichi Osozawa
sg9	Japan: Kyushu, Tanega‐shima	*Stylogomphus ryukyuanus ryukyuanus*	LC546154	LC546214	LC546274	LC546334	28‐May‐12	Soichi Osozawa
sg14	N Taiwan:Yangmingshan	*Stylogomphus shirozui shirozui*	LC546155	LC546215	LC546275	LC546335	24‐May‐13	Soichi Osozawa
sg20b	Japan: Ryukyu, Okinawa‐jima, Motobu	*Stylogomphus ryukyuanus asatoi*	LC546156	LC546216	LC546276	LC546336	02‐Jul‐12	Soichi Osozawa
sg21b	Japan: Ryukyu, Okinawa‐jima, Yona	*Stylogomphus ryukyuanus asatoi*	LC546157	LC546217	LC546277	LC546337	29‐Jun‐10	Soichi Osozawa
sg23a	Japan: Ryukyu, Tokuno‐shima, Mikyo	*Stylogomphus ryukyuanus ryukyuanus*	LC546158	LC546218	LC546278	LC546338	05‐Jul‐13	Soichi Osozawa
sg24	Japan: Ryukyu, Amami Oshima, Yuwan	*Stylogomphus ryukyuanus ryukyuanus*	LC546159	LC546219	LC546279	LC546339	08‐Jul‐13	Soichi Osozawa
sg33	China: HongKong, TaiPo	*Stylogomphus chunliuae*	LC546161	LC546221	LC546281	LC546341	12‐May‐15	Soichi Osozawa
sg35	Japan: Honshu, Miyagi, Sendai	*Stylogomphus suzukii*	LC546163	LC546223	LC546283	LC546343	15‐Jul‐15	Soichi Osozawa
sg36a	Japan: Kyushu, Kagoshima, Kinko	*Stylogomphus ryukyuanus ryukyuanus*	LC546164	LC546224	LC546284	LC546344	31‐Jul‐15	Hidetoshi Sugita
sg37	Japan: Honshu, Miyagi, Sendai	*Trigomphus melampus*	LC546165	LC546225	LC546285	LC546345	12‐May‐16	Soichi Osozawa
sg41	Japan: Shikoku, Kagawa, Zentsuji	*Trigomphus citimus tabei*	LC546166	LC546226	LC546286	LC546346	20‐May‐16	Soichi Osozawa
sg42	Japan: Shikoku, Tokushima, Mima	*Trigomphus interruptus*	LC546167	LC546227	LC546287	LC546347	22‐May‐16	Soichi Osozawa
sg44	Japan: Honshu, Miyagi, Zao	*Lanthus fujiacus*	LC546168	LC546228	LC546288	LC546348	28‐May‐16	Soichi Osozawa
sg45	Japan: Honshu, Miyagi, Zao	*Davidius nanus*	LC546169	LC546229	LC546289	LC546349	28‐May‐16	Soichi Osozawa
sg53	Japan: Honshu, Miyagi, Zao	*Davidius moiwanus moiwanus*	LC546170	LC546230	LC546290	LC546350	01‐Jul‐16	Soichi Osozawa
ag3	Japan: Ryukyu, Okinawa‐jima, Yona	*Asiagomphus amamiensis okinawanus*	LC546171	LC546231	LC546291	LC546351	24‐Jun‐10	Soichi Osozawa
ag8	Japan: Chiba, Inzai	*Asiagomphus pryeri*	LC546172	LC546232	LC546292	LC546352	23‐May‐13	Soichi Osozawa
ag10	Japan: Ryukyu, Ishigaki‐jima, Omoto	*Asiagomphus yayeyamensis*	LC546173	LC546233	LC546293	LC546353	07‐Jun‐13	Takehiko Yamanaka
ag11a	N Taiwan:Yangmingshan	*Asiagomphus hainanensis*	LC546174	LC546234	LC546294	LC546354	15‐Jun‐13	Soichi Osozawa
ag22	Korea: Busan	*Asiagomphus melaenops*	LC546175	LC546235	LC546295	LC546355	20‐May‐14	Soichi Osozawa
ag35a	Japan: Ryukyu, Amami Oshima, Tatsugo	*Asiagomphus amamiensis amamiensis*	LC546176	LC546236	LC546296	LC546356	01‐Jun‐14	Koji Tanimura
ag33b	Japan: Honshu, Miyagi, Sendai	*Asiagomphus melaenops*	LC546177	LC546237	LC546297	LC546357	20‐Jun‐13	Soichi Osozawa
ag39B	Japan: Honshu, Miyagi, Sendai	*Sieboldius albardae*	LC546178	LC546238	LC546298	LC546358	01‐Jul‐16	Soichi Osozawa
ag40	Japan: Shikoku, Kagawa, Manno‐ike	*Asiagomphus melaenops*	LC546179	LC546239	LC546299	LC546359	23‐May‐16	Soichi Osozawa
ag41	Japan: Shikoku, Kagawa, Manno‐ike	*Asiagomphus melaenops*	LC546180	LC546240	LC546300	LC546360	24‐May‐16	Soichi Osozawa
ant4a	Japan: Ryukyu, Okinawa‐jima, Yona	*Polycanthagyna melanictera*	LC136181	LC546241	LC546301	LC546361	23‐Jun‐13	Soichi Osozawa
ant12	Japan: Honshu, Miyagi, Sendai	*Anotogaster sieboldii*	LC546182	LC546242	LC546302	LC546362	05‐Aug‐13	Takayasu Ito
ant5	Japan: Ryukyu, Amami Oshima, Yuwan	*Anotogaster sieboldii*	LC546183	LC546243	LC546303	LC546363	08‐Jul‐13	Soichi Osozawa
ant17	Japan: Ryukyu, Okinawa‐jima, Iji	*Anotogaster sieboldii*	LC546184	LC546244	LC546304	LC546364	27‐Jun‐14	Kyoji Osozawa
ant1	N Taiwan:Yangmingshan	*Anotogaster klossi* (*Anotogaster sieboldii*)	LC546185	LC546245	LC546305	LC546365	07‐Jun‐13	Soichi Osozawa
ant13	China: Zhejiang	*Anotogaster klossi* (*Anotogaster sieboldii*)	LC546186	LC546246	LC546306	LC546366	02‐Oct‐12	Akira Mishima
ag34a	Japan: Honshu, Miyagi, Sendai	*Tanypteryx pryeri*	LC546187	LC546247	LC546307	LC546367	01‐Jun‐14	Soichi Osozawa
ag31a	Japan: Honshu, Miyagi, Sendai	*Epiophlebia superstes*	LC546188	LC546248	LC546308	LC546368	26‐May‐14	Soichi Osozawa
mat4	Japan: Ryukyu, Okinawa‐jima, Yona	*Matrona japonica* (*Matrona basilaris*)	LC546189	LC546249	LC546309	LC546369	20‐Jun‐13	Soichi Osozawa
mat32	Japan: Kyushu, Tanega‐shima	*Atrocalopteryx atrata*	LC546190	LC546250	LC546310	LC546370	06‐Jul‐12	Soichi Osozawa
mat33	Japan: Honshu, Miyagi, Sendai	*Calopteryx japonica* (*Matrona basilaris japonica*)	LC546191	LC546251	LC546311	LC546371	20‐Jul‐10	Soichi Osozawa
rh1	S Taiwan:Tairuko	*Rhipidolestes aculeatus*	LC546192	LC546252	LC546312	LC546372	06‐Aug‐12	Soichi Osozawa
rh3	Japan: Ryukyu, Ishigaki‐jima, Miyara‐gawa	*Rhipidolestes aculeatus*	LC546193	LC546253	LC546313	LC546373	04‐May‐10	Soichi Osozawa
rh4	Japan: Ryukyu, Iriomote‐jima, Urauchi‐gawa	*Rhipidolestes aculeatus*	LC546194	LC546254	LC546314	LC546374	30‐Apr‐10	Soichi Osozawa
rh11b	Japan: Shikoku, Tokushima, Kotsu‐yama	*Rhipidolestes hiraoi*	LC546195	LC546255	LC546315	LC546375	28‐Jul‐11	Soichi Osozawa
rh15	S Taiwan:Hualien	*Rhipidolestes aculeatus*	LC546196	LC546256	LC546316	LC546376	01‐Jun‐13	Soichi Osozawa
rh16a	N Taiwan:Yangmingshan	*Rhipidolestes aculeatus*	LC546197	LC546257	LC546317	LC546377	07‐Jun‐13	Soichi Osozawa
rh17c	Japan: Ryukyu, Okinawa‐jima, Oura‐gawa	*Rhipidolestes okinawanus*	LC546198	LC546258	LC546318	LC546378	19‐Jun‐13	Soichi Osozawa
rh18	Japan: Ryukyu, Okinawa‐jima, Motobu	*Rhipidolestes okinawanus*	LC546199	LC546259	LC546319	LC546379	19‐Jun‐13	Soichi Osozawa
rh19c	Japan: Ryukyu, Okinawa‐jima, Yona	*Rhipidolestes shozoi*	LC546200	LC546260	LC546320	LC546380	20‐Jun‐13	Soichi Osozawa
rh21b	Japan: Ryukyu, Tokashiki‐jima	*Rhipidolestes okinawanus*	LC546201	LC546261	LC546321	LC546381	24‐Jun‐13	Soichi Osozawa
rh21B	Japan: Ryukyu, Tokuno‐shima, Sasontsuji‐dake	*Rhipidolestes amamiensis*	LC546202	LC546262	LC546322	LC546382	06‐07‐13	Soichi Osozawa
rh23B	Japan: Ryukyu, Amami Oshima, Yuwan	*Rhipidolestes amamiensis*	LC546203	LC546263	LC546323	LC546383	08‐07‐13	Soichi Osozawa
rh24b	Japan: Kyushu, Kagoshima, Kobayashi	*Rhipidolestes yakusimensis*	LC546204	LC546264	LC546324	LC546384	29‐Jun‐14	Kunihiko Matsuhira
rh26a	Japan: Ryukyu, Tokuno‐shima, Kobaru coast	*Rhipidolestes amamiensis*	LC546205	LC546265	LC546325	LC546385	05‐07‐12	Takuya Murata
rh27	Japan: Kyushu, Yaku‐shima	*Rhipidolestes yakusimensis*	LC546206	LC546266	LC546326	LC546386	25‐Jun‐12	Takuya Murata
rh28	Japan: Kyushu, ShimoKoshiki‐jima	*Rhipidolestes asatoi*	LC546207	LC546267	LC546327	LC546387	03‐Jul‐12	Takuya Murata
rh13	China: HongKong, TaiPo	*Agriomorpha fusca*	LC546208	LC546268	LC546328	LC546388	28‐May‐13	Soichi Osozawa
sg38	Japan: Honshu, Miyagi, Sendai	*Orthetrum japonicum*	LC546209	LC546269	LC546329	LC546389	12‐May‐16	Soichi Osozawa

Furthermore, for specific Odonata data related to taxonomy, refer to Kim et al. (2014) for Korean Odonata, Kiyoshi (2008) for Cordulegastridae, Kiyoshi and Sota (2006) for Gomphidae, and Ware et al. (2017) for North American Gomphidae. Data on Petaluridae can be found in Ware et al. (2014). Additionally, you can find visual references in colored picture books, including Ishida et al. (1988), Ozono et al. (2007, 2012), Sugimura et al. (2008), Yangmingshan National Park Management Office (1996; for northern Taiwan), and Wilson (2003; for Hong Kong).

### 2.2 DNA extraction and polymerase chain reaction amplification, sequence alignment

For the present study, we obtained a total of 58 sequence datasets, consisting of the mitochondrial COI gene (3P; 795 bp), COII gene (5P; 548 bp), 16S rRNA (517 bp), and the nuclear 28S rRNA gene (825 bp), totaling 2685 bp, as outlined in Table 2 (Table 1; in comparison to recent studies). For further analytical details, including the primer sets used, refer to Osozawa, Sato, et al. (2017), which also included a total of 30 datasets available in the present analyses, in addition to the previously published data in Osozawa and Wakabayashi (2015).

The COI‐3P and COII‐5P gene data overlap with the tRNA‐Leu region, as described in Osozawa, Sato, et al. (2017), and note that data for the corresponding region are rarely available in GenBank/DDBJ. However, COI‐5P data, amplified using the LCO‐HCO universal primers (Folmer et al., 1994; Osozawa, Takáhashi, et al., 2017), are frequently found in GenBank/DDBJ but are not compatible with our COI‐3P sequence.

In BEAST v1.10.4, the analysis of combined genes, including mitochondrial COI, COII, and 16S rRNA, along with the nuclear 28S rRNA, can be performed simply by configuring partitions for these genes. Concatenation of these genes and re‐partitioning are not necessary (Osozawa, 2023).

Our selected mitochondrial genes exhibit a high base substitution rate and higher resolution compared to nuclear genes, making them suitable for studying very old ages without experiencing mutation saturation, as discussed in Osozawa et al. (2012); Osozawa, Sato, et al. (2017), Osozawa and Wakabayashi (2022, updated in 2023), and Osozawa (2023). This is in contrast to the challenges posed by paralogs observed in nuclear genes (Gabaldón & Koonin, 2013). Notably, Ho et al. (2005) found it improbable that the apparent decline in rates over time could be attributed to mutational saturation, and we propose the absence of mutation saturation (Osozawa & Wakabayashi, 2022, updated in 2023) and Osozawa (2023). To further explore this, we examined the relationship between pairwise distance and the number of transitions or transversions for each gene (Figure 2), applying the MEGA11 function (Tamura et al., 2021).

### 2.3 Phylogenetic analyses by BEAST v1.10.4

Apart from the calibration functional descriptions below, refer to section 2.2. “Phylogenetic analyses by BEAST v1.10.4: Tutorial” in Osozawa (2023). We reprint as Appendix S1: Phylogenetic analyses by BEAST v1.10.4: Tutorial‐Summary.

BEAST v1.10.4 incorporates a calibration function within its associated software, BEAUti v1.10.4 (Bayesian Evolutionary Analysis Utility). The calibration process in BEAUti entails specifying a time of the most recent common ancestor (tMRCA) for the ingroup taxa, effectively establishing a crown node date for a particular clade. Consequently, we can establish a prior for the specific clade and its crown age. Note that this tMRCA definition aligns with the approach used in BEAST 2.7.5 and is analogous to the minimum age applied in MCMCTree 4.9 and MrBayes v3.2.7. Neither a maximum age nor root age constraint is imposed in BEAST v1.10.4 and BEAST 2.7.5, as discussed in more detail in Osozawa (2023). For a comprehensive understanding of our calibration methods, refer to the details presented below.

### 2.4 Quaternary geologic event: Ryukyu continental islands formed at 1.55 Ma for BEAST v1.10.4 analyses

Osozawa et al. (2012) illustrated how the back‐arc spreading of the Okinawa Trough led to the formation of the Ryukyu Islands, resulting in their separation from the Chinese mainland. This sea‐floor spreading process initiated the islands' separation approximately 1.55 ± 0.15 Ma, and this isolation has persisted over time. The rapid subsidence required for each island's creation led to their simultaneous isolation from the Chinese mainland and from one another, primarily due to the formation of the Okinawa Trough and other significant seaways such as the Tsushima and Taiwan straits. These straits, along with some minor ones in specific instances, are thought to have acted as barriers to migration, thus triggering vicariance.

**FIGURE 3:**
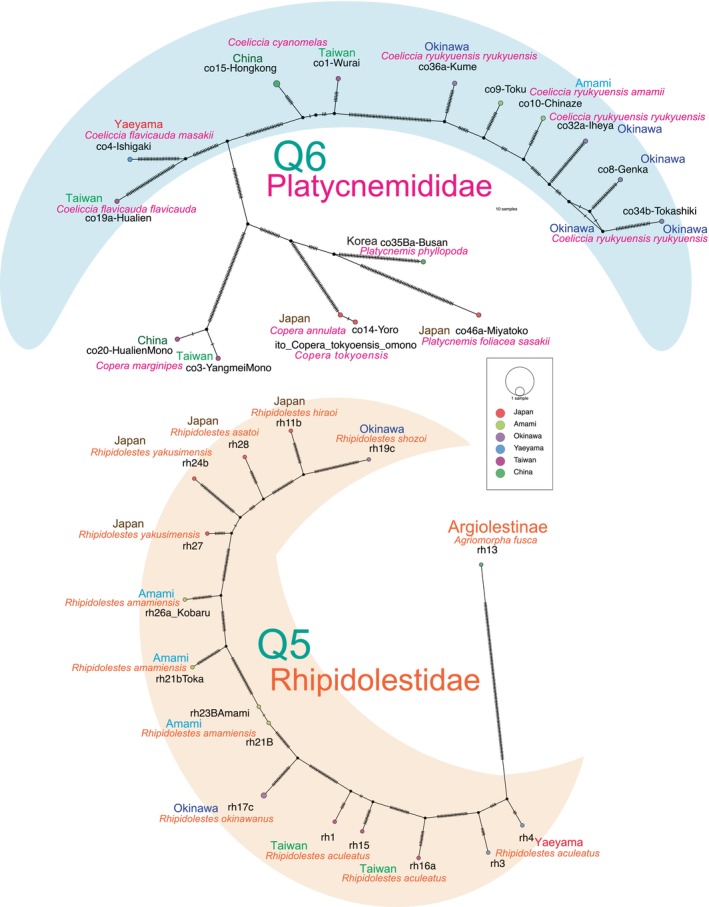
Haplotype networks for Platycnemididae (Q6) and Rhipidolestidae (Q5). Haplotypes on the background half‐moon represent a clade of Q6 and Q5, respectively, indicating vicariance associated with adaptive radiation. Dispersal is not considered. Refer to Figure 1 for corresponding details.

Considering these geological factors, the date of 1.55 Ma serves as a robust calibration point (tMRCA) applied in BEAST v1.10.4 for six Odonata clades: *Stylogomphus* (Gomphidae; calibration point Q1), *Asiagomphus* (Gomphidae; Q2; note that *A. hainanensis* in China was not collected, and sympatric *A. pryeri* and *A. coreanus* were not included in the *Asiagomphus* ingroup), *Chlorogomphus* (Chlorogomphoidea; Osozawa & Wakabayashi, 2015; Q3), *Anotogaster* (Cordulegastridae; Kiyoshi, 2008; Osozawa et al., 2013; Q4), *Rhipidolestes* (Rhipidolestidae; Q5; with the Chinese species not collected), and *Coeliccia* (Platycnemididae; Osozawa, Sato, & Wakabayashi, 2017; Q6), as outlined in Figure 1 and Table 3. To visually represent the Q5 (Rhipidolestidae) and Q6 (Platycnemididae) clades, we created haplotype network diagrams in Figure 3 applying PopART version 1.7 (Population Analysis with Reticulate Trees; Leigh & Bryant, 2015).

**TABLE 3:** Fossil and geological event calibrations.

Calibration point	Fossil	Family	Infraorder‐suborder	Order	Ingroup clade	Kohli et al. (2016)	Formation	System	Stage	tMRCA (ma)	Method	Paleontological reference	Geological reference
A	Unnamed	Machilidae		Archaeognatha	Machilidae		Gaspé	Devonian	Emsian	400.45 ± 7.15	Correlation	Labandeira et al. (1988)	Parks (1931)
A1 (not applied)	*Gigamachilis triassicus* ^a^	Machilidae		Archaeognatha	Machilidae		Kalkschieferzone	Triassic	Ladinian	239.51 ± 0.15	Correlation	Montagna et al. (2017)	Montagna et al. (2017)
B	*Delitzschala bitterfeldensis* ^a^	Spilapteridae^a^		Palaeodictyoptera^a^	Palaeoptera		Bitterfeld/Delitzsch	Carboniferous	Namurian	319.5 ± 6.5	Correlation	Brauckmann & Schneider (1996)	Brauckmann and Schneider (1996)
C	Trace fossil			Ephemeroptera^a^	Ephemeroptera		Wamsutta	Carboniferous	Gzhelian	301.3 ± 2.4	Correlation	Knecht et al. (2011)	Lyons and Sproule (2018)
D	*Huangiopterum lodevense* ^a^	Huangiopteridae^a^		Odonatoptera	Odonata		Salagou Formation	Permian	Artinskian	282.86 ± 0.13	U–Pb dating	Prokop et al. (2015)	Michel et al. (2015)
D	Saxonagrion minutus^a^	Saxonagrionidae^a^		Odonatoptera^a^	Odonata		Salagou Formation	Permian	Artinskian	282.86 ± 0.13	U–Pb dating	Nel et al. (1999)	Michel et al. (2015)
D1 (not applied)	Egg insertion scars		Zygoptera	Odonata	Zygoptera (stem)		Madygen	Triassic	Ladinian‐Carnian	234.5 ± 7.5	Lacking	Moisan et al. (2012)	Lacking
D2 (not applied)	*Triassolestodes asiaticus* ^a^	Triassolestidae^a^	Epiprocta	Odonata	Epiprocta	(1) crown Odonata	Madygen	Triassic	Ladinian	242 ± 5	Lacking	in Kohli et al. (2016)	Lacking
E	*Liassophlebia* ^a^	Liassophlebiidae^a^	Epiprocta	Odonata	*Epiophlebia superstes* (stem)		Lilstock	Triassic	Rhaetian	204.9 ± 3.6	correlation	Kelly and Nel (2018)	established
E1 (not applied)	*Liassophlebia* ^a^	Liassophlebiidae^a^	Epiprocta	Odonata	*Epiophlebia superstes* (stem)	(3) crown Epiprocta	Keuper	Jurassic	Hettangian	200.3 ± 1.0	Correlation	in Kohli et al. (2016)	Established
E2 (not applied)	*Dorsettia sinica* ^a^	Campterophlebiidae^a^	Isophlebiida^a^	Odonata	Odonata		Badaowan	Jurassic	Hettangian‐Sinemurian	196.05 ± 5.25	*Waagenoperna*	Zheng et al. (2016)	Pan et al. (2013)
F	*Sinacymatophlebia mongolica* ^a^	Cymatophlebiidae^a^	Aeshnoptera^a^	Odonata	Anisoptera	(4) crown Anisoptera	Daohugou Biota	Jurassic	Oxfordian	160.54 ± 0.99	U–Pb dating	Nel and Huang (2009)	Liu et al. (2012)
G	*Prohemeroscopus jurassicus* ^a^	Hemeroscopidae^a^	Aeshnoptera^a^	Odonata	Chlorogomphoidea		Solnhofen limestone	Jirassic	Tithonian	148.5 ± 3.6	Ammonite	Bechly et al. (1998)	Stuttgart (2007)
G1	*Protolindenia wittei* ^a^	Petalurida^a^	Anisoptera	Odonata	Petaluridae (stem)		Solnhofen limestone	Jirassic	Tithonian	148.5 ± 3.6	Ammonite	Nel et al. (1998)	Stuttgart (2007)
G2	*Jurahemiphlebia haeckeli* ^a^	Hemiphlebiidae^a^	Zygoptera	Odonata	Zygoptera		Solnhofen limestone	Jirassic	Tithonian	148.5 ± 3.6	Ammonite	Bechly (2019)	Stuttgart (2007)
G3 (not applied)	*Enteropia mongolica*	Hemiphlebiidae^a^	Zygoptera	Odonata	Zygoptera		Ulan Malgait	Jirassic	Tithonian	148.5 ± 3.6	Lacking	Dollman et al. (2018)	Lacking
G4 (not applied)	*Anglopetalura magnifica* ^a^	Petalurida	Anisoptera	Odonata	Petaluridae		Purbeck limestone	Cretaceous	Berriasian	142.4 ± 2.6	Correlation	Coram and Nel (2009)	Established
G5 (not applied)	*Argentinopetala archangelskyi* ^a^	Petalurida^a^	Anisoptera	Odonata	Petaluridae		Anfiteatro de Ticó	Cretaceous	Aptian	119.65 ± 0.45	Ar‐Ar dating	Petrulevicius and Nel (2003)	in Petrulevicius and Nel (2003)
H	*Proterogomphus renateae* ^a^	Proterogomphidae^a^	Anisoptera	Odonata	Gomphidae (stem)	(6) crown Gomphidae	Solnhofen limestone	Jurassic	Tithonian	148.5 ± 3.6	Ammonite	Bechly et al. (1998)	Stuttgart (2007)
H1 (not applied)	*Liogomphus yixianensis* ^a^	Gomphidae	Anisoptera	Odonata	Gomphidae		Jehol Biota	Cretaceous	Hauterivian	130.7 ± 1.4	Ar‐Ar dating	Ren and Guo (1996)	He et al. (2006)
H2 (not applied)	*Rudiaeschna limnobia* ^a^	Rudiaeshnidae^a^	Anisoptera	Odonata	Aeshnidae		Jehol Biota	Cretaceous	Hauterivian	130.7 ± 1.4	Ar‐Ar dating	Ren and Guo (1996)	He et al. (2006)
H3	*Mesocordulia* (*Guocordulia*) *boreala* ^a^	Corduliidae	Anisoptera	Odonata	Corduliidae		Jehol Biota	Cretaceous	Hauterivian	130.7 ± 1.4	Ar‐Ar dating	Ren and Guo (1996)	He et al. (2006)
H4 (not applied)	Unnamed^a^	Macromiidae	Anisoptera	Odonata	*Epophthalmia elegans* (stem)		Lagerstätte Enspel	Paleogene	Oligocene Chattian	24.675 ± 0.115	Ar‐Ar dating	Brockhaus et al. (2020)	Mertz et al. (2007)
H5 (not applied)	*Epophthalmia zotheca* ^a^	Macromiidae	Anisoptera	Odonata	*Epophthalmia elegans* (stem)	(8) crown Macromiidae	Shanwang	Neogene	Miocene Burdigalian	16.45 ± 0.45	Correlation	in Kohli et al. (2016)	Rocˇek et al. (2011)
H6 (not asigned)	*Epophthalmia biordinata* ^a^	Macromiidae	Anisoptera	Odonata	*Epophthalmia elegans* (stem)	(8) crown Macromiidae	Latah	Neogene	Miocene	NOT asigned	K‐Ar	in Kohli et al. (2016)	Gray and Kittleman (1967)
H7 (not applied)	*Cordulagomphus* ^a^	Proterogomphidae^a^	Anisoptera	Odonata	Gomphidae		Santana	Cretaceous	Aptian‐Cenomonian	112.5 ± 1.25	Correlation	Petrulevicius et al. (2012)	Martill (2007)
H8 (not applied)	*Burmaeshna azari* ^a^	Burmaeshnidae^a^	Aeshnoptera^a^	Odonata	Aeshnidae		Burmese amber	Cretaceous	Cenomanian	98.79 ± 0.62	U–Pb dating	Huang et al. (2017)	Shi et al. (2012)
H9 (not asigned)	*Gomphaeschna inferna* ^a^	Aeshnidae	Anisoptera	Odonata	Aeshnidae	(5) crown Aeshnidae	Zaza (not Zara)	Cretaceous	Berriasian	142.4 ± 2.6	Lacking	Bechly et al. (2001)	Lacking
I	*Palaeolibellula zherikhini* ^a^	Libellulidae	Anisoptera	Odonata	Libellulidae		Zhirkindek	Cretaceous	Turonian	91.85 ± 2.05	lacking	Fleck et al. (1999)	Lacking
I1 (not applied)	*Urolibellula eocenica* ^a^	Urolibellulidae^a^	Anisoptera	Odonata	Libellulidae		Green River	Paleogene	Yepresian	51.25 ± 0.31	Ar‐Ar dating	Grande (1980) Zeiri et al. (2015)	Smith et al. (2003)
I2 (not applied)	*Tauriphila*? *cerestensis* ^a^	Libellulidae	Anisoptera	Odonata	Libellulidae	(10) crown Libellulidae	Créste	Paleogene	Oligocene Rupelian	28.465 ± 5.435	Correlation	Nel and Paicheler (1993)	Ducreux et al. (1985)
J	*Calopteryx japonica*	Calopterygidae	Zygoptera	Odonata	*Calopteryx japonica* +		Shimabara graben	Quaternary	Pleistocene	1.76 ± 0.22	Fission track	Esaki and Asahina (1957)	Okaguchi and Otsuka (1980)
K	*Sinocalopteryx shangyongensis* ^a^	Calopterygidae	Zygoptera	Odonata	Calopterygidae		Yunnan	Paleogene	Eocene Yepresian	51.9 ± 4.1	Correlation	Lin et al. (2010)	Lin et al. (2010)
K1 (not applied)	*Calopteryx andancensis* ^a^	Calopterygidae	Zygoptera	Odonata	*Calopteryx japonica* (stem)		Ardèche	Neogene	Miocene Turolian	7.0165 ± 1.6835	Correlation	Nel and Brisac (1994)	Nel and Brisac (1994)
X	*Gulou carpenteri* ^a^			Plecoptera	Plecoptera		Tupo Formation	Carboniferous	Pennsylvanian	311.05 ± 12.15	Correlation	Béthoux et al. (2011)	Legendre et al. (2015)
Q1	Geological event	Gomphidae	Anisoptera	Odonata	*Stylogomphus ryukyuanus*		Ryukyu	Quaternary	Pleistocene Calabrian	1.55 ± 0.15	Biostratigraphy		Osozawa et al. (2012)
Q2	Geological event	Gomphidae	Anisoptera	Odonata	*Asiagomphus* spp.		Ryukyu	Quaternary	Pleistocene Calabrian	1.55 ± 0.15	Biostratigraphy		Osozawa et al. (2012)
Q3	Geological event	Cordulegastridae	Anisoptera	Odonata	*Anotogaster* spp.		Ryukyu	Quaternary	Pleistocene Calabrian	1.55 ± 0.15	Biostratigraphy	Osozawa et al. (2013)	Osozawa et al. (2012)
Q4	Geological event	Chlorogomphoidea	Anisoptera	Odonata	*Chlorogomphus* spp.		Ryukyu	Quaternary	Pleistocene Calabrian	1.55 ± 0.15	Biostratigraphy	Osozawa & Wakabayshi (2015)	Osozawa et al. (2012)
Q5	Geological event	Rhipidolestidae	Zygoptera	Odonata	*Rhipidolestes* spp.		Ryukyu	Quaternary	Pleistocene Calabrian	1.55 ± 0.15	Biostratigraphy		Osozawa et al. (2012)
Q6	Geological event	Platycnemididae	Zygoptera	Odonata	*Coeliccia* spp.		Ryukyu	Quaternary	Pleistocene Calabrian	1.55 ± 0.15	Biostratigraphy	Osozawa, Kanai, et al. (2021)	Osozawa et al. (2012)

*Note*: Refer to Figure 1 for the precise application of fossil calibration points on dated tree.

^a^ Extinct.

### 2.5 Geological evaluation of fossil dates for BEAST v1.10.4 analyses

Misof et al. (2014), much like Montagna et al. (2019), as listed in Table 1, developed a comprehensive insect timetree, which included three representatives of Odonata, including *E. superstes*. They estimated the Archaeognatha basal node at 440 Ma applying BEAST v.1.8. They explicitly mentioned that their calibration date adhered closely to the protocol proposed by Parham et al. (2012), which, according to the present authors, including a structural geologist, is deemed geologically robust.

We concur with their approach, which emphasizes reviewing the published geological age and the stratigraphic range of the fossil, and aligning the data with the most up‐to‐date standard global geochronology. Furthermore, they stress the importance of evaluating the quality of stratigraphic information for each fossil. Notably, they made exceptions for certain amber deposits, excluding most of them from their analysis. For instance, the Baltic amber was dated to the Lutetian period using Ar–Ar dating at 44.3 ± 0.4 Ma (Wappler, 2005), while the Dominican amber was assigned to the Aquitanian period (ranging from 23.03 to 20.44 Ma; 21.735 ± 1.295 Ma; Iturralde‐Vinent & MacPhee, 1996). They emphasized that “biostratigraphic information has been updated and adapted to the current geological time scale,” following the most recent International Chronostratigraphic Chart established by the International Commission on Stratigraphy (Cohen et al., 2013; using v 2023/09 updates).

Kohli et al. (2016) conducted an extensive examination of numerous crown dates available for Odonata fossil calibration (as presented in their Table 2). However, it is essential to acknowledge that there is some degree of uncertainty surrounding fossil ages (not fossil identifications). Therefore, we conducted a reevaluation of the geological constraints linked to the fossil locations. We selected several pre‐Quaternary calibration dates, taking into account their geological reliability, which included factors such as whether the fossil ages were constrained by modern and more precise radioisotopic dating or by stratigraphical correlation.

Table 3 provides a summary of our fossil calibration, with further details available in Appendix S2: fossil calibration.

### 2.6 Picking the target tree with rate data

The target tree, fully calibrated by both fossil and geological event dates, and encompassing both pre‐Quaternary and Quaternary dates, is presented in Figure 1.

We are revisiting a time‐dependent phenomenon, specifically the exponential increase in phylogenetic base substitution rates in a relatively recent timeframe. To achieve this, we have initiated the following projects: “FigTree is designed as a graphical viewer of phylogenetic trees and as a program for producing publication‐ready figures. In particular, it is designed to display summarized and annotated trees produced by BEAST.” FigTree v1.4.4 provides the capability to visualize base substitution rates on each node, enabling us to explore time dependencies. Unfortunately, other graphical user interface applications like BEAST 2.7.5 (Bouckaert et al., 2019), as well as command‐line applications, such as MCMCTree in PAML (4.9e 2017; Yang, 2007) and MrBayes (v3.2.72019; Ronquist et al., 2012), do not calculate and display the rate in FigTree output figure. Therefore, these applications are not suitable for our current research objectives.

In FigTree v1.4.4, we can obtain outputs for the posterior age (“Node ages”), posterior probability (“posterior”), and “rate median” (mean of three rates for three branches at a specific node; not constant due to the application of a relaxed clock model; Drummond et al., 2006). These values are displayed at each node in Figure 1. Accordingly, we created a diagram (Figure 1 inset) illustrating the base substitution rate (“rate median,” displayed at each node) versus age (“Node age,” displayed at each node). This diagram includes a power trendline, its equation, and an *R* ^2^ value of 0.0266.

**FIGURE 4:**
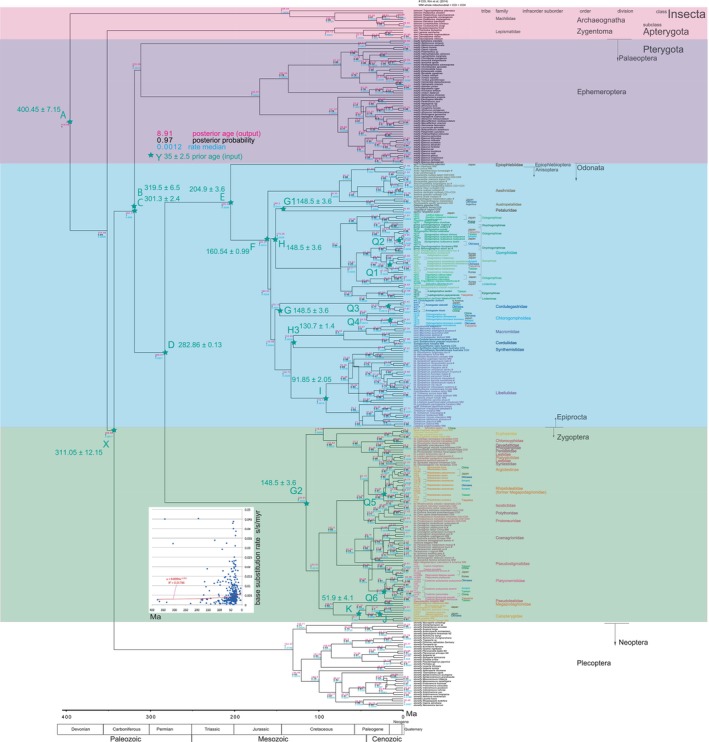
BI Tree Calibrated Solely by Pre‐Quaternary Fossil Dates, Utilizing BEAST v1.10.4. Observe that point K is indeed a fossil calibration point, but its date falls within the Quaternary. Both point K and points Q1–Q6 have the time to most recent common ancestor (tMRCA) set to default and have not been assigned specific dates. Notably, this tree does not incorporate any Quaternary node ages, with the minimum node age being 5.05 Ma, which closely aligns with the maximum root age of 5.8 Ma in Figure 5. Inset: Base substitution rate versus age diagram. It is essential to note that the rates depicted here are significantly slower than those shown in Figure 1, often falling into a single‐digit range.

**FIGURE 5:**
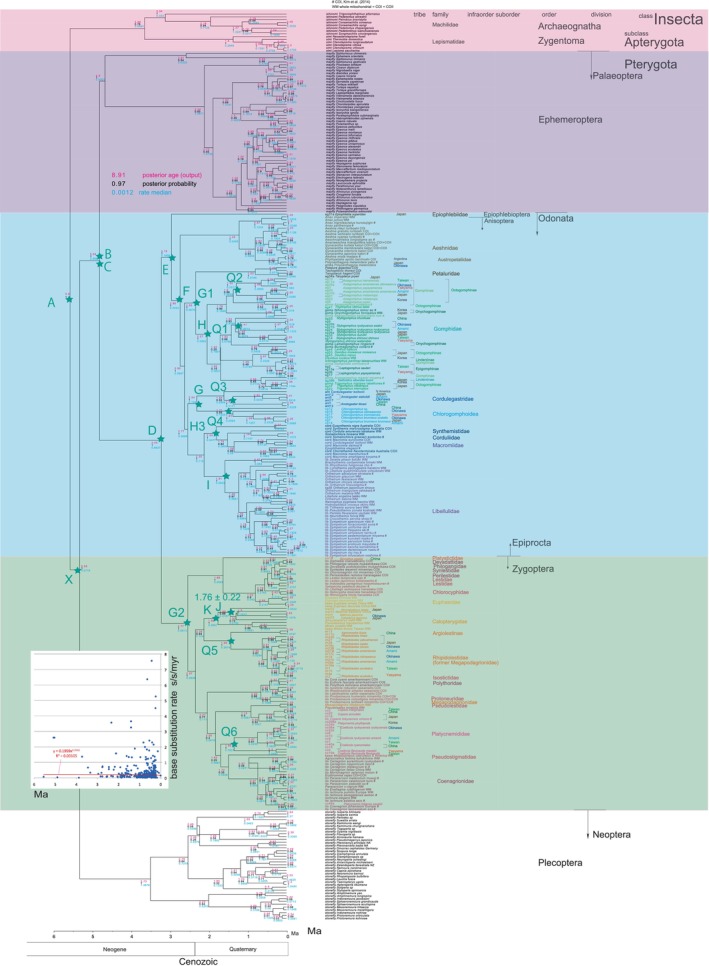
BI tree calibrated solely by Quaternary dates, employing BEAST v1.10.4. It is important to recognize that point K is a fossil calibration point, and its date falls within the Quaternary, making it part of the current calibration. Calibration points A to I have the time to most recent common ancestor (tMRCA) set to default values, without specific dates assigned. Remarkably, this tree lacks the extended timescales seen in Figure 1, with the root age estimated at only 5.8 Ma, in stark contrast to the 393.39 Ma age in Figure 1. Inset: Base substitution rate versus age diagram. Take note that the rates displayed here are notably higher than those in Figures 1 and 4.

To examine a time‐dependent base substitution rate influenced by the Quaternary calibration (Ho et al., 2005; Papadopoulo et al., 2010), we constructed a dated tree solely calibrated by the Quaternary dates of Q1–Q6 and J (Figure 4). We also generated a tree solely calibrated by the pre‐Quaternary dates of A to I, K, and X, excluding Q1–Q6 and J (Figure 5).

## 3 RESULTS

### 3.1 Dated tree

The posterior probabilities at the basal or crown nodes of Archaeognatha, Zygentoma, Palaeoptera, Ephemeroptera, Odonata, Epiprocta, Anisoptera, Zygoptera, and Plecoptera are all 1, except for one instance where the probability is 0.99. This high probability indicates the reliability of the current topology (Figure 1).

The crown age of Insecta was estimated to be 393.39 Ma, with Archaeognatha (Apterygota) identified as the oldest lineage within Insecta. Zygentoma (Apterygota) follows as the second oldest lineage. Apterygota is paraphyletic, and Palaeoptera is monophyletic. Ephemeroptera (Pterygota) is sister to Odonata (Pterygota), and this split occurred around 313.7 Ma. Palaeoptera is sister to Neoptera (Plecoptera), with their differentiation estimated to have occurred at 332.43 Ma.

Epiprocta, which encompasses Anisoptera and Epiophlebioptera, is sister to Zygoptera and diverged approximately 284.24 Ma. *Epiophlebia superstes*, a member of the Epiophlebiidae within the Epiophlebioptera order and sister to Anisoptera, is a long‐standing lineage with a considerably extended terminal branch. The age of this species is estimated to be around 205.75 million years, indicative of its profound evolutionary history.

The Anisoptera major clade encompasses each of its family clades, determined through the establishment of the most recent common ancestor (MRCA) and its corresponding ingroup species (also referred to as a clade or monophyrum). These family clades include Aeshnidae, Petaluridae, Cordulegastridae + Chlorogomphoidea, Gomphidae, Macromiidae + Corduliidae + Synthemistidae, and Libellulidae.

Aeshnidae, found to be sister to the remaining Aeshnoptera, dates back to an age as old as 161.94 Ma. It further diverged as a sister to the resting Anisoptera at 158.49 Ma.

Petaluridae is sister to Gomphidae, with their divergence occurring approximately 152.3 Ma. Petaluridae + Gomphidae is sister to the remainder of the resting Anisoptera.

Cordulegastridae + Chlorogomphoidea, emerged around 147.8 Ma, is sister to the resting Macromiidae + Corduliidae + Synthemistidae, with the latter group acting as the sister to Libellulidae, dating back to approximately 134.38 Ma.

In the analysis of Zygoptera, each selected zygopteran family formed a distinct clade, totaling 19 clades. The only exception to this pattern is *Mesopodagrion tibetanum*, which is discussed separately. The largest damselfly, *Megaloprepus caerulatus*, is sister to Coenagrionidae.

A multifurcation is observed among the species inhabiting the Rykyu‐Taiwan‐Japan Island region, including Chinese species. This complex pattern, reflecting a history of vicariance, began around 1.55 Ma and is observed in four ingroup species for Anisoptera (Q1–Q4) and two ingroup species for Zygoptera (Q5 and Q6; see Figure 3). The differentiation of *Matrona* and *Calopteryx* species is dated to 1.76 Ma, as calibrated by the point “J.”

### 3.2 Base substitution rates

The base substitution rate versus age diagram reveals that the rate is not constant, as seen in the application of the relaxed clock model. Instead, it exhibits an exponential increase since approximately 20 Ma, as depicted in the equation shown in the inset (Figure 1 inset). The mean rate, which averaged 0.0205 s/s/my between ca. 200 and 20 Ma, exponentially escalated to 0.334 s/s/my during the Pleistocene, leading to vicariant speciation in *Rhipidolestes*. The actual maximum rate observed was 0.76 s/s/my in Plecoptera during the Miocene.

Since our timetree covers a time span extending from 393.39 million years post‐Silurian, calibrated up to 400.45 million years at point A, the base substitution rate versus age diagram captures extremely ancient variations in substitution rates. Another noteworthy observation is a mild peak in the rate between the Carboniferous and Permian periods, with rates of 0.0553 s/s/my at the crown node of Paleoptera (313.7 Ma) and 0.0412 s/s/my at the crown node of Odonata (284.24 Ma).

Figure 2 demonstrates that even mitochondrial genes with rapid base substitution rates, as discussed in Osozawa, Sato, et al. (2017), do not exhibit saturation toward ancient time periods.

### 3.3 Impact of the Quaternary calibration on dated tree

The haplotype networks shown in Figure 3 support the ingroup or clade setting for the Quaternary calibration.

The phylogenetic tree calibrated exclusively using pre‐Quaternary dates is depicted in Figure 4, and it notably exhibits a topology that aligns with the fully calibrated tree displayed in Figure 1. However, the estimated ages for nodes in Figure 4 are considerably older than those in Figure 1, approximately 20 million years older than the calibration point dates in Figure 4. Furthermore, the base substitution rates in Figure 4 are significantly slower, measuring less than 0.05 substitutions s/s/my. In this example tree in Figure 4, five branches are collapsed or compressed, resulting in a distortion of “space–time.” When constructing Figure 1, we encountered this distorted tree and had to reset and recalculate.

Conversely, the phylogenetic tree calibrated solely using Quaternary dates is presented in Figure 5 and exhibits a topology consistent with the fully calibrated tree in Figure 1. However, in Figure 5, the estimated node ages are notably younger, with the root age being only 5.8 Ma (resulting in every node age being less than 5.8 Ma). The base substitution rates in Figure 5 are substantially higher, reaching up to 7.56 s/s/my. Similar to Figure 4, there are two instances where branches in this tree appear to be collapsed or compressed, indicating inaccuracies in Figure 5.

## 4 DISCUSSION

### 4.1 Dated tree

It is important to note that our application of mostly mitochondrial genes successfully estimated ancient divergence times, and there is no evidence of mutation saturation (Figure 2). Our results support the paraphyly of Apterygota and the monophyly of Archaeognatha, Zygentoma, Pterygota, Palaeoptera, Ephemeroptera, Odonata, Epiprocta, Zygoptera, and each Odonata family. Ephemeroptera is sister to Odonata, Epiprocta is sister to Zygoptera, and Palaeoptera is sister to Neoptera (Figure 1; c.f., Figures 3 and 4; Table 1). These findings are consistent with Misof et al. (2014) and Montagna et al. (2019).

Regarding the issue of overestimated ancient node ages in Misof et al. (2014), where their table S4 contains only minimum age information, and Montagna et al. (2019) compared to our Figure 1, Klopfstein (2021) argued, “Their node dating approaches have a credibility problem: different studies using the same molecular data and even the same sets of fossils regularly arrive at drastically different age estimates. A major reason for these differences is well‐known: even well‐dated and firmly placed fossils can only provide a minimum age for a specific node.” Another potential contributing factor to the overestimation of node ages is their reliance on calibrations solely by pre‐Quaternary dates, as illustrated in our Figure 4. It is essential to consider that their dating may be inaccurate, while our Figure 1, which incorporates both Quaternary and pre‐Quaternary calibrations, is more reliable.

Epiprocta is established as the sister to Zygoptera, while Epiophlebioptera is identified as the sister to Anisoptera. Within Anisoptera, Aeshnidae emerges as the sister to the remaining Anisoptera. Moving further, Petaluridae is recognized as the sister to Gomphidae, and the combined clade of Petaluridae + Gomphidae acts as the sister to the remaining Anisoptera. Additionally, Cordulegastridae is determined to be the sister to Chlorogomphoidea, and Cordulegastridae + Chlorogomphoidea is established as the sister to the remaining Anisoptera. Within this remaining group, Macromiidae + Corduliidae + Synthemistidae is the sister to Synthemistidae. These relationships are consistent with Kohli et al. (2021) and Suvorov et al. (2022). These studies tend to overestimate node ages, and this could be attributed to factors such as the utilization of maximum age and the reliance on calibrations solely based on pre‐Quaternary data.

We presented a timetree for the East Asian *Coeliccia* damselfly, with details on vicariance provided in Osozawa, Sato, et al. (2017). This timetree was calibrated exclusively using the Quaternary date of geological events, set at 1.55 ± 0.15 Ma. However, a significant issue emerged during this analysis, as the calculated basal node age (root age) was found to be much younger than what would be considered reasonable. This chronological problem is not unique to the *Coeliccia* damselfly but also appeared in the analyses of other East Asian Odonata timetrees, such as the *Anotogaster* dragonfly (Osozawa et al., 2013) and the *Chlorogomphus* dragonfly (Osozawa & Wakabayashi, 2015), as well as in other East Asian insects like the *Papilio* butterflies (Osozawa et al., 2013), *Mycalesis* butterfly (Osozawa, Takáhashi, et al., 2015), *Pyrocoelia* firefly (Osozawa, Oba, et al., 2015), *Cicindela* tiger beetle (Osozawa, Fukuda, et al., 2016; Osozawa, Ogino, et al., 2016), *Ypthima* butterfly (Osozawa, Takáhashi, et al., 2017), *Platypleura* cicada (Osozawa, Shiyake, et al., 2017), Ryukyu endemic five cicada group (Osozawa, Kanai, et al., 2021), and carabid ground beetle (Osozawa, Ogino, et al., 2016). Note that while these papers successfully describe extensive vicariance resulting from the isolation of islands from the Chinese continent, it has become evident that the inclusion of pre‐Quaternary calibration points, in addition to Quaternary data, is necessary for achieving more precise dating in these analyses.

*Epiophlebia superstes* is a constituent of the Epiophlebioptera clade, with the sister group being the Anisopteromorpha, represented here by the Liassophlebiidae, a member of the stem group of the Anisoptera clade. Both of these groups collectively constitute the extant representatives of the Epiprocta major clade. Remarkably, *E. superstes* has persevered as an independent lineage, with an age as ancient as 205.75 Ma. *Epiophlebia* spp. have been identified in isolated regions encompassing the Japanese islands, China (the same species has also been reported in North Korea; Gunther et al., 2013), and the Himalayas. Despite this broad distribution, indications suggest that the vicariance in these areas has been relatively mild, implying that the isolation occurred recently and is not directly related to the age of this lineage (Busse et al., 2012). A parallel situation can be observed in the Araucariaceae conifer, an ancient clade with very young taxa in recent times (Escapa & Catalano, 2013).

Petaluridae, encompassing *Tanypteryx pryeri* and *Petalura gigantea*, was originally dated at 152.3 Ma, placing them in the earliest Cretaceous period. However, Ware et al. (2014) proposed that the entire Petalurida group, which includes Petaluridae, is an ancient clade with roots dating back to the Triassic (>201.3 Ma). This extended timeframe for their diversification may be linked to the breakup of the supercontinent Pangaea. As detailed in our methods section (Appendix S2), it is noteworthy that the Pangaea breakup and the initiation of the Atlantic Ocean were recorded in the Santana Formation, occurring between 125 and 100 Ma (Martill, 2007; Table 3). This timeframe is notably more recent than the previously estimated crown age of 152.3 Ma for Petaluridae. Moreover, it is essential to acknowledge that the history of Pangaea's breakup is considerably more intricate, as we have explored in the context of mammalian evolution (Osozawa, 2023).

At the family level, differentiation within the extant Anisoptera occurred between 161.94 and 134.38 Ma, while for the extant Zygoptera, it took place from 116.4 to 3.85 Ma (Figure 1). Species‐level differentiation in Odonata occurred after 34.92 Ma, mainly during the Paleogene and Neogene periods. The endemic species and subspecies, which were calibrated with points Q1 to Q6 at 1.55 Ma, differentiated during the Quaternary. It is important to note that in the dated trees created by Kohli et al. (2021) and Suvorov et al. (2022), species‐level differentiation is predominantly from the Paleogene, with some instances extending into the Cretaceous, and these estimates are now known to be overestimated due to the use of maximum age and reliance on calibrations solely based on pre‐Quaternary data.

Yu and Bu (2011) conducted a cladistic analysis of damselflies and demonstrated that Mesopodagrion and Rhipidolestes belong to different families, effectively splitting the former “Megapodagrionidae” into two distinct families, Megapodagrionidae and Rhipidolestidae. The two distinct clades of “Megapodagrionidae” in Figure 1 are indicative of this subdivision.

### 4.2 Quaternry vicariance increased biodiversity

Calibration points Q1 to Q6, representing *Stylogomphus*, *Asiagomphus*, *Anotogaster*, *Chlorogomphus* (Osozawa & Wakabayashi, 2015), *Rhipidolestes*, and *Coeliccia* (Osozawa, Sato, et al., 2017), were set at the 1.55 ± 0.15 Ma geologic event. Each of these multi‐furcations or polytomies in the phylogenetic tree (Figure 1) and each of the haplotype network patterns (Figure 3) signify the emergence of multiple endemic species. The isolation of islands resulting from the opening of the Okinawa trough is a physical process, but it is evident that this process has significantly contributed to an increase in biodiversity. While such isolation may lead to severe bottlenecks for endemic species, it is intriguing that this bottleneck effect does not align with the observed low genetic diversity and low nucleotide substitution rates found in this case, which contrasts with Zhai et al. (2017).

Calibration point J, representing *Calopteryx* and *Matrona*, was calibrated based on a fossil date of 1.76 ± 0.22 Ma. The divergence between *Calopteryx japonica* (found in Japan) and *Matrona japonica* (located in Amami‐Okinawa; although *Matrona basilaris* in the Taiwan specimen could not be amplified, data from China were applied) appears to be a consequence or possibly a precursor to the 1.55 Ma vicariance event, as depicted in Figure 1.

In general, the process of back arc spreading, leading to the formation of continental islands, has the potential to trigger vicariance events that significantly contribute to increased biodiversity. There are numerous back arc basins in the western Pacific Ocean, some of which remain active, including the Okinawa trough. The notable high diversity of terrestrial organisms observed on continental islands, separated from continental landmasses due to sea‐floor spreading of this nature, is likely a direct result of the physical isolation of these islands stemming from the rifting process, and the subsequent vicariance that ensues.

Conversely, oceanic islands are also created through back arc spreading, where volcanic edifices emerge in the ocean. However, these islands do not initially host terrestrial life because they surface above sea level as a result of ongoing volcanic activity. In such cases, the islands acquire their initial terrestrial species through dispersal from other landmasses. The species diversity associated with oceanic islands is more likely to increase through vicariance after the initial colonization via dispersal (Osozawa, Ito, et al., 2021; Osozawa, Kanai, et al., 2021; Osozawa, Ogino, et al., 2016).

We demonstrated that lotic damselflies tend to undergo vicariant speciation, in contrast to their lentic counterparts, as described in Osozawa, Sato, et al. (2017). A similar finding was reported by Letsch et al. (2016). However, it is important to note that within the Octogomphinae subfamily, there is no such tendency observed between the lotic *Davidius* and lentic *Trigomphus*.

Libellulidae, however, is predominantly composed of lentic species, where vicariance may be minimal, indicated by the similarity in genetic sequences between island populations, resulting in lower species diversity. An exception to this pattern is *Sympetrum pedemontanum*, which is a lotic species within the *Sympetrum* clade of Libellulidae. This lotic tendency is also observed in some species of mostly lentic Coenagrionidae damselflies.

### 4.3 Increasing base substitution rate and biodiversity

Applying only the pre‐Quaternary calibration resulted in the generation of very slow base substitution rates, as illustrated in the inset of Figure 4. Conversely, when solely the Quaternary calibration was applied, it led to the emergence of very rapid base substitution rates, as depicted in the inset of Figure 5. The use of either of these calibration methods in isolation yielded invalid rates. However, the age versus rate diagram in the inset of Figure 1 remains a valid consideration, as it reflects the time‐dependent nature of these rates and is of significant value.

The base substitution rates exhibited a shift from a relatively constant slower rate before approximately 20 Ma to a higher rate, especially during the Quaternary, as illustrated in the inset of Figure 1. This phenomenon contradicts the molecular clock hypothesis (which posits a relatively constant rate over time, as per Ho, 2008) and makes rate dating unsuitable for Insecta evolution. Instead, the rates have been observed to exponentially increase toward more recent times, as indicated in the inset of Figure 1. This phenomenon is not merely an apparent one, and this conclusion is supported by the absence of gene saturation, as shown in Figure 2.

An increase in base substitution rate could reasonably be expected to contribute to greater biodiversity, as exemplified by the vicariance‐induced biodiversity observed in the case of the 1.55 Ma event mentioned earlier. Vicariance can be at least partly explained by the adaptive radiation of *Rhipidolestes amamiensis*, resulting in the formation of distinct clades (Figures 1 and 3). However, it is also important to consider an alternative perspective. The greater apparent biodiversity in geologically recent times might be influenced by the greater availability and preservation of, as well as the consequently increased number of studies conducted on, recent geological sections (as discussed in Rohde & Muller, 2005). Molecular phylogenetic analyses, in conjunction with paleobiological studies (Buatois & Mángano, 2018; Neige, 2015; Sahney et al., 2010), can be employed to test these alternative hypotheses (c.f., Barrier et al., 2001; Jønsson et al., 2012; Nakamura et al., 2021).

A possible driving factor behind the exponential increase in evolutionary rates and the likely rise in biodiversity toward the present time is extensive adaptive radiation (c.f., Ho et al., 2011). This could be attributed to the onset of glacial and interglacial cycles, coupled with environmental changes during the Quaternary period starting approximately 2.58 Ma, as discussed in Osozawa (2023). Following Ho et al. (2011), human mitochondrial DNA reflects adaptive changes in response to climatic variations (Mishmar et al., 2003; Ruiz‐Pesini et al., 2004). Adaptive radiation within Murinae has been partly associated with positive selection and genomic changes (Roycroft et al., 2021). Genome analyses also suggest that the adaptive radiation of Heliconiini butterflies, the same target of Brower (1994), led to both phenotypic and genotypic variance (Cicconardi et al., 2023; see their Figure 1). Note that the Quaternary glaciations might have been triggered by the expansion of C4 land grasses and the evolution of sea diatoms, as this process led to increased carbon fixation, subsequently resulting in a decrease in atmospheric CO_2_ concentration, as proposed by Taira (2007). The expansion of C4 grasses was a global phenomenon, encompassing regions such as North America and South America, and it initiated in the Oligocene and extended into the late Miocene, persisting to the present day. This expansion has relevance to the current glacial–interglacial period, although with some time lag, as highlighted by Cerling et al. (1997).

Although the available data plots were somewhat limited, we observed another peak in the base substitution rate during the Carboniferous to Permian period, as indicated in the inset of Figure 1. This peak may be analogous to the Late Paleozoic Ice Age, as discussed by Montañez et al. (2016), Rolland et al. (2019), and Rosa and Isbell (2020). While glacial episodes in Earth's history are known to be influenced by factors such as landmass configuration, including the Gondwana supercontinent, it is possible that feedback from biological developments also played a role in initiating this glaciation. The Late Paleozoic glaciation is believed to have been triggered by the proliferation of terrestrial plants, specifically ferns, leading to the formation of thick coal layers during the Carboniferous period, as proposed by Franks et al. (2014). This process had the effect of increasing carbon fixation, which in turn effectively reduced atmospheric CO_2_ levels, as previously suggested by Taira (2007) and corroborated by Montañez et al. (2016) and Rolland et al. (2019).

## 5 CONCLUSION

Constant evolution rates through time, which form the foundation of the strict molecular clock model, do not apply in the case of Paleoptera, highlighting a notable bias in the molecular clock hypothesis. The current biodiversity may be the outcome of a relatively recent, exponential increase in base substitution rates. This could be a reflection of the initiation of Quaternary glacial and interglacial cycles linked to substantial climatic changes. Additionally, another rate peak was identified in the Carboniferous to Permian period, which is similarly associated with the late Paleozoic icehouse.

## AUTHOR CONTRIBUTIONS

**Soichi Osozawa:** Conceptualization (lead); data curation (lead); formal analysis (lead); funding acquisition (lead); investigation (lead); methodology (lead); project administration (lead); resources (lead); software (lead); supervision (lead); validation (lead); visualization (lead); writing – original draft (lead); writing – review and editing (lead). **André Nel:** Conceptualization (supporting); data curation (supporting); formal analysis (supporting); funding acquisition (supporting); investigation (supporting); methodology (supporting); project administration (supporting); resources (supporting); software (supporting); supervision (supporting); validation (supporting); visualization (supporting); writing – original draft (supporting); writing – review and editing (supporting).

## FUNDING INFORMATION

This research was partly supported by Grants‐in‐Aid for Scientific Research Japan, “Extrusion Wedge of the Sambagawa High P‐T Metamorphic Rocks,” grant number 20540441.

## CONFLICT OF INTEREST STATEMENT

The author declares that there are no competing interests.

## Supporting information


Appendix S1.

Appendix S2.

Data S1.

## Data Availability

All relevant data are included in the manuscript. Supplementary data in Table 1 are available at GenBank/DDBJ, and accession numbers are provided in Table 2. The xml file, generated by BEAUti for running the BEAST platform software, includes all the applied sequence data and can be found in the supplementary material.
