# Supplementary material for: Paleopteran molecular clock: Time drift and recent acceleration
Source: Ecol Evol. 2024 Sep 18;14(9):e70297. doi: 10.1002/ece3.70297 (PMC11410561; doi:10.1002/ece3.70297)
Supplement: Supplementary file 1 — Appendix S1. [file ECE3-14-e70297-s002.docx]

**Phylogenetic analyses by BEAST v1.10.4: Tutorial-Summary**

Reprinting after Osozawa (2023).

The protocol has evolved with the newer versions of BEAST v1.8 (and v1.10.4) from BEAST v1.7 (Drummond et al., 2012). It is recommended to use the latest version of BEAST v1.10.4.

To construct the Bayesian inference (BI) tree (Figs. 2 and 3), the BEAST software package was used. The analysis involves running BEAUti v1.10.4, BEAST, TreeAnnotator v1.10.4, and FigTree v1.4.4, in that order. It is important to download the BEAGLE Library before using the BEAST software platform. Tracer v1.6 was utilized to check the calculation status and estimate the mean base substitution rate.

In BEAUti, the following software settings were used:

Partitions: The fasta files were loaded using the "Import Data" or plus button. COI, COII, 16S rRNA, and the nuclear 28S rRNA data constitute four partitions, appearing in the Partition box.

Taxa: The taxa were added as an ingroup by selecting the plus button in BEAUti. In the left screen of BEAUti, the Taxon Set was configured with monophyletic boxes checked for all taxa, while the stem box was checked on a case-by-case basis. The right screen displayed the included monophyletic taxa, representing specific clades.

Tips: Default.

Sites: Substitution Model: HKY (Hasegawa, Kishino and Yano) model, Base frequencies: Empirical, Site Heterogeniety Model: Gamma, Number of Gamma Categories: 4, Partition into codon positions: Off. The GTR model generates similar topology.

Clocks: Clock Type: Uncorrected relaxed clock, Relaxed Distribution: Lognormal.

Trees: Tree Prior: Speciation: Yule Process. See details in Gernhard (2008) and Heath (2022).

Priors: tmrca (time of MRCA; time of most recent common ancestor) was input from the calibration point date as Prior Distribution: Normal, and as the Mean and Standard deviation.

MCMC: Length of chain: 10,000,000. Increased length such as 20,000,000 only spends time and unaffected on output.

Running BEAST was done by incorporating xml input file made by BEAUti. The tree files were input into TreeAnnotator, and the consequent tree was drawn by FigTree v1.4.4.
