# Supplementary material for: Paleopteran molecular clock: Time drift and recent acceleration
Source: Ecol Evol. 2024 Sep 18;14(9):e70297. doi: 10.1002/ece3.70297 (PMC11410561; doi:10.1002/ece3.70297)
Supplement: Supplementary file 2 — Appendix S2. [file ECE3-14-e70297-s003.docx]

**Fossil calibration**

We incorporated a total of 16 fossil calibration points labeled from A to K and X (as depicted in Fig. 1), which resulted in a robust time calibration reaching as far back as 400 Ma. It is noteworthy that the Devonian calibration point A was specifically associated with Archaeognatha, while the Carboniferous calibration points B and C were linked to Paleoptera and Ephemeroptera, respectively, based on our own extensive bibliographic survey and reevaluation, as outlined also in Fig. 1.

Each label provided below corresponds to the calibration points in the subsequent BEAST v1.10.4 analysis, which can be found also in Fig. 1. These fossil dates are listed in descending order of their ages, from A to J, with K and X excluded.

A: The oldest fossil, attributed to Archaeognatha, was recovered from the Devonian period (Emsian; 407.6 ~ 393.3 Ma; 400.45 ± 7.15 Ma), originating in Gaspé, Québec, Canada, as reported by Parks in 1931 (Labandeira et al., 1988). We have adopted this date as calibration point A for Archaeognatha.

A1 (not applied): *Gigamachilis triassicus* gen. et sp. nov. (Archaeognatha) was discovered in the Kalkschieferzone (239.51 ± 0.15 Ma) of Monte San Giorgio (a UNESCO World Heritage Site, situated between Switzerland and Italy) (Montagna et al., 2017, 2019). These authors noted that *G. triassicus*, possessing morphological features indicative of its placement within the extant archaeognathan ingroup Machilidae, provides an age for the modern lineages during the Middle Triassic.

B: *Delitzschala bitterfeldensis* Brauckmann & Schneider, 1996 (Palaeodictyoptera, Paleoptera), was reported from the Namurian stage (319.5 ± 6.5 Ma) in Bitterfeld / Delitzsch, Northeastern Germany.

C: Knecht et al. (2011) provided evidence of a complete body impression of a flying insect from the Late Carboniferous Wamsutta Formation in Massachusetts, USA. This discovery represents the oldest trace fossil of Pterygota, specifically Ephemeroptera (mayfly). We have considered the Gzhelian stage, which falls within the range of 298.9 ~ 303.7 Ma (Lyons and Sproule, 2018, in accordance with Cohen et al., 2013, with a refined estimate of 301.3 ± 2.4 Ma), as the date for calibration point C.

D: The oldest stem lineages of the Odonata clade are found in the Permian period (represented by fossils *Huangiopterum lodevense*, as described by Prokop et al., 2015, and *Saxonagrion minutus*, as detailed by Nel et al., 1999, both from Lodève in Southern France). The Octon Member of the Salagou Formation, where these fossils were found, has been dated using the U-Pb method, yielding an age of 282.86 ± 0.13 Ma (Michel et al., 2015).

D1 (not applied): Misof et al. (2014) applied a calibration date derived from fossil egg insertion scars found in host plants, which were associated with damselflies (Moisan et al., 2012) from the Madygen Formation in Kyrgyzstan (Voigt et al., 2006). This led them to consider the root age of Odonata to be in the Triassic period (Ladinian-Carnian; 234.5 ± 7.5 Ma). However, this conflicts with the previously mentioned date D of 282.86 ± 0.13 Ma. It is important to note that egg insertion can be attributed to various insect groups beyond Odonata *sensu stricto*, including the Triassic Protozygoptera and other more inclusive Odonatoptera clades, which could produce similar features.

D2 (not applied): Kohli et al. (2016) introduced a node calibration (1) for crown Odonata, involving *Triassolestodes asiaticus* from the Madygen Formation. Based on our experience in Vietnam (Osozawa et al., 2015c), where geological survey methods were influenced by both Russian and French approaches, we propose a need for reconsideration of the Ladinian (and Carnian) age in Kyrgyzstan.

E: A *Liassophlebia* fossil was recently discovered in the Lilstock Formation in England, dating back to the Rhaetian stage (Kelly and Nel, 2018; 204.9 ± 3.6 Ma). We have adopted the age of 204.9 ± 3.6 Ma as the stem age for *Epiophlebia superstes*, corresponding to the crown age of Epiprocta.

E1 (not applied): Another Liassophlebia fossil was found in the Bayreuth Formation, located between the Keuper (one of the three Triassic strata in Germany) and the Gryphaeensandstein Formation (Kohli et al., 2016). The stratigraphy is well-established, and it is correlated with the Hettangian stage, the earliest portion of the Jurassic, with an age range of 201.3 ~ 199.3 Ma (200.3 ± 1.0 Ma), which is slightly younger than the 204.9 ± 3.6 Ma mentioned earlier. It is worth to note that the Liassophlebiidae are not directly related to the Epiophlebiidae; their relationships remain somewhat uncertain. However, the Liassophlebiidae, belonging to Heterophlebioptera, are more closely related to the Anisoptera *sensu stricto* than to the Epiophlebiidae, which occupy a more inclusive position (Bechly, 1996). Therefore, the date of 204.9 ± 3.6 Ma may be equivalent to the stem age of Anisoptera (Pananisoptera), with the oldest record being Liassogomphidae from the Toarcian, dating to 183.0 Ma. In Fig. 1, calibration point E, in fact, represents the stem of Epiophlebioptera as well as Anisoptera.

E2 (not applied): *Dorsettia sinica* (Campterophlebiidae, Isophlebiida) was discovered in the Badaowan Formation in Inner Mongolia (Zheng et al., 2016) and biostratigraphically estimated to be Hettangian-Sinemurian, approximately 196.05 ± 5.25 Ma, which is younger than the aforementioned date of 204.9 ± 3.6 Ma. The Isophlebiida are part of the clade Isophlebioptera, currently considered the sister group of the clade (Epiophlebioptera + Anisopteromorpha), known from the Jurassic (*Sogjutella*, Huang et al., 2018).

F: *Sinacymatophlebia mongolica* (Cymatophlebiidae; Aeshnoptera; Nel and Huang, 2009) was discovered in the Daohugou Biota, Inner Mongolia. The age of the strata, which also provides constraints on the age of feathered dinosaurs, has been determined as 160.54 ± 0.99 Ma (Liu et al., 2012). This U-Pb age is considered the crown age of Anisoptera. A U-Pb age of approximately 160 Ma was also obtained from the Daohugou beds by Liu et al. (2006).

G: The Solnhofen limestone, known for its *Archaeopteryx* fossils, has yielded *Prohemeroscopus jurassicus*. The Hemeroscopidae are considered part of the stem group of the Chlorogomphoidea (Beckly et al. 1998). The biostratigraphic age, as determined using ammonite fossils, corresponds to the Tithonian stage (152.1 ~ 145.0 Ma; specifically 148.5 ± 3.6 Ma; Stuttgart, 2007).

G1: *Protolindenia wittei* (Petalurida) was found in the Solnhofen limestone (Nel et al., 1998; 148.5 ± 3.6 Ma) and is adopted as the stem age for Petaluridae.

G2: The Tithonian Solnhofen limestone has also yielded Zygoptera fossils, including *Jurahemiphlebia haeckeli* (Beckly, 2019), with the calibration point G2 set at 148.5 ± 3.6 Ma. *Hemiphlebia mirabilis* (Hemiphlebiidae; Zygoptera), known as the ancient greenling, is endemic to the southeastern end of Australia and Tasmania (only 18S rRNA available in GenBank/DDBJ). The oldest Hemiphlebiidae fossil is *Jurahemiphlebia haeckeli* Bechly 2019 from the Solnhofen limestone, corresponding to G2.

G3 (not applied): *Enteropia mongolica* (Hemiphlebiidae) was found in the Tithonian Ulan Malgait Formation of Mongolia (Dollman et al., 2018). In our experience of field surveys in Mongolia (Osozawa et al., 2008), however, the Tithonian age may require reconsideration.

G4 (not applied): The upper Purbeck Limestone in England has yielded insect fossils, including Petalurida dragonflies like *Anglopetalura magnifica* (Nel, 2009). The age is stratigraphically correlated with the Berriasian stage, which represents the lowermost Cretaceous (145 ~ 139.8 Ma; specifically 142.4 ± 2.6 Ma), making it younger than the previously mentioned 148.5 ± 3.6 Ma.

G5 (not applied): The oldest known crown Petaluridae is *Argentinopetala archangelskyi* Petrulevicius & Nel 2003 from the Lower Cretaceous of Argentina, with Ar-Ar dating indicating an age of 119.65 ± 0.45 Ma (Petrulevicius and Nel, 2003). However, BEAUti cannot simultaneously consider the stem box (G1) or not (G5) for the same Petaluridae clade, and we have applied G1.

H: *Proterogomphus renateae* (Proterogomphidae) was reported from the Solnhofen limestone (Bechly et al., 1988), and we have considered it as the stem age for the Gomphidae clade.

H1 (not applied): *Liogomphus yixianensis* (Gomphidae) from China; specifically, the Jehol Biota horizon was dated using the Ar-Ar method applied to intercalated silicic tuff, resulting in an age of 130.7 ± 1.4 Ma (He et al., 2006).

H2 (not applied): *Rudiaeschna limnobia* (Rudiaeshnidae, stem group of Aeshnidae); and H3: *Mesocordulia boreala* (Corduliidae Gomphomacromiinae), described from the Jehol Biota in northern China (Ren and Guo, 1996). It is important to note that *Mesocordulia* May 1991 (subgenus of the extant genus *Neocordulia*) has priority over *Mesocordulia* Ren & Guo 1996. Therefore, we suggest the replacement name of *Guocordulia* for *Mesocordulia* Ren & Guo 1996.

H4 (not applied): The oldest Macromiidae were described from the Lagerstätte Enspel, Germany (Brockhaus et al., 2020), and the Ar-Ar ages were determined to be 24.79 ~ 24.56 Ma (Mertz et al., 2007). Since we included only one species of Macromiidae, these dates, along with any dates younger than H3 (130.7 ± 1.4 Ma), are not applicable.

H5 (not applied): *Epophthalmia zotheca* (Macromiidae) was found in Shanwang, Shandong, China (in Kohli et al., 2016), and these strata are considered to be time-correlative with the European MN5 mammalian unit (16.45 ± 0.45 Ma; Roček et al., 2011).

H6 (not applied): *Epophthalmia biordinata* (Macromiidae) was found in the Latah Formation, Washington, USA (in Kohli et al., 2016), but the dating was done using the traditional K-Ar method only (Gray and Kittleman, 1967), while the Columbia River Basalts were dated using the Ar-Ar method, indicating an age younger than 16.6 Ma (Barry et al., 2013).

H7 (not applied): The Santana Formation in northeastern Brazil, near the Atlantic margin, is known for well-preserved fish fossils and also includes fossils of *Cordulagomphus*, a Proterogomphidae dragonfly (Petrulevicius et al., 2012). Although no reliable index fossils are present and no volcanic rock is interbedded for radiometric dating, the age is considered to be coeval with the breakup of the supercontinent Gondwana and the initiation of the Atlantic Ocean. It may be Aptian, Albian, or possibly Cenomanian in the Cretaceous, representing a rather loosely-constrained date (approximately 125 ~ 100 Ma; Martill, 2007).

H8 (not adopted): Burmese amber yielded Aeshnoptera (the total group of Aeshnidae; Huang et al. 2017), and the U-Pb age is 98.79 ± 0.62 Ma (Shi et al., 2012), which was not adopted due to its younger age.

H9 (not assigned): *Gomphaeschna inferna* was reported from the Zaza Formation (not Zara Formation) in Buryatia, Russia (Bechly et al., 2001), but chronological data are lacking.

I: Libellulidae were reported from the Upper Cretaceous of Kazakhstan (Fleck et al., 1999), but the age was not constrained by geological evidence (Gratshev and Zherikhin, 2003). Therefore, the "Turonian" calibration point was not assigned. Kohli et al. (2016) did not use it to calibrate the crown group node Libellulidae because the phylogenetic position of this fossil species within the family Libellulidae is not resolved, and it might instead represent a stem Libellulidae. However, we tentatively calibrated the crown group Libellulidae at 91.85 ± 2.05 Ma (Turonian).

I1 (not applied): The Green River Formation in the USA yielded well-preserved insect fossils, including Libellulidae and Zygoptera (Grande, 1980). Ar-Ar dating applied to the silicic tuff within the formation yielded ages of 53.5 to 48.5 Ma (weighted average age of 51.25 ± 0.31 Ma; Smith et al., 2003). This fossil was later identified as Urolibellulidae, the sister group of the extant Libellulidae (Zeiri et al., 2015).

I2 (not applied): Libellulidae were also found at the fossil locality of Céreste in southern France (Nel and Paicheler, 1993). The fossils included the *Lethe* butterfly (Nel et al., 1993; Pfretzschner, 1998) mentioned in Osozawa et al. (2017a). The assigned age is loosely constrained as middle Oligocene (33.9 ~ 23.03 Ma; Ducreux et al., 1985), representing a large relative uncertainty. Therefore, this age is not adopted for calibration, also considering the younger age of the Green River Formation. Note that these Oligocene fossils represent the crown Libellulidae, while the taxa from the Green River Formation only represent the sister group of the Libellulidae, including the Turonian fossil.

J: Wing fossils of extant *Calopteryx japonica* (= *Matrona basilaris japonica*) and *Oligoaeschna pryeri* (= *Sarasaeschna pryeri*; no reliable COI-COII sequence data were obtained) were found in the fluvial to deltatic strata of the Beppu-Shimabara graben (Esaki and Asahina, 1957), which is located in the northeastern extension and branch of the Okinawa trough (cf., Osozawa K. et al. 2016). The fission track age of the dacitic tuff breccia within these strata is 1.76 ± 0.22 Ma, and the strata are magnetically reversed, considered to fall within the Matsuyama Chron (2.58 ~ 0.77 Ma; Okaguchi and Otsuka, 1980), consistent with the above fission track age. Note that the J point is included in the Quaternary calibration.

K: The oldest fossil Calopterygidae, *Sinocalopteryx shangyongensis*, was reported from the Eocene of Yunnan, China (Lin et al., 2010; 51.9 ± 4.1 Ma).

K1 (not applied): *Calopteryx andancensis* Nel & Brisac 1994, the oldest known *Calopteryx*, was found in the Upper Miocene of France (Turolian, 8.7 to 5.333 Ma; 7.0165 ± 1.6835 Ma; Tortonian ~ Messinian). However, BEAUti only allows for setting the crown age of K, so the stem box is not checked for this *Calopteryx* clade.

X: The wing venation of *Gulou carpenteri* exhibits character states diagnostic of the order Plecoptera, and constitutes stem-Plecoptera (Béthoux et al., 2011). The fossil was found in the Tupo Formation (Pennsylvanian; Late Carboniferous; northern China) and is referred to the Qilianshan biota, which also yielded *Sinomeganeura huangheensis* (Ren et al., 2008). The Pennsylvanian age is estimated through insect fossil correlation, as radiometric dating is not available (Legendre et al., 2015).

**References only for appendix S2.**

Huang D, Cai C-y, Nel A, Bechly G. 2017. A new dragonfly family from the mid Cretaceous Burmese amber (Odonata: Aeshnoptera: Burmaeshnidae). Cretaceous Research, 78, 8–12.

Liu Y, Liu Y, Zhang H. 2006. LA-ICPMS zircon U-Pb dating in the Jurassic Daohugou Beds and correlative strata in Ningcheng of Inner Mongolia. Acta Geologica Sinca, 80, 133–142.

May M. 1991. A review of the genus Neocordulia, with a description of *Mesocordulia* subgen. nov. and of *Neocordulia griphus* spec. nov. from Central America, and a note on *Lauromacromia* (Odonata: Corduliidae). Folia Entomológica Mexicana, 82, 17-67.

Osozawa S, Tsolmon G, Majigsuren U, Sereenen J, Niitsuma S, Iwata N, Pavlis T, Jahn BM. 2008. Structural evolution of the Bayanhongor region, west-central Mongolia. Journal of Asian Earth Sciences, 33, 337–352.

Osozawa S, Vuong NV, Tich VV, Wakabayash J. 2015. Reactivation of a collisional suture by Miocene transpressional domes associated with the Red River and Song Chay detachment faults, northern Vietnam. Journal of Asian Earth Sciences, 105, 252–269.

Pfretzschner HU. 1998. Ein weiteres exemplar von Lethe? Corbieri Nel 1993 (Lepidoptera, Satyridae) aus dem Unter- Oligozan von Cereste (Sud-Frankreich). Palaontologische Zeitschrift, 72, 56–64.

Ren D, Nel A, Prokop J. 2008. New early griffenfly, *Sinomeganeura huangheensis* from Late Carboniferous of northern China (Meganisoptera: Meganeuridae). Insect Systematics & Evolution, 38, 223-229.

Roček, Z., Dong, L., Přikryl, T., Sun, C., Tan, J., Wang, Y. 2011. Fossil frogs (Anura) from Shanwang (Middle Miocene; Shandong Province, China). Geobios,. 44, 499–518.

Voigt S, Haubold H, Meng S, Krause D, Buchantschenko J, Ruckwied K. Götz AE. 2006. Die Fossil-Lagerstätte Madygen: ein Beitrag zur Geologie und Paläontologie der Madygen-Formation (Mittel-bis Ober-Trias, SW-Kyrgyzstan, Zentralasien). Hallesches Jahrbuch für Geowissenschaften, 2, 85–119.
